# Differences between kinematic synergies and muscle synergies during two-digit grasping

**DOI:** 10.3389/fnhum.2015.00165

**Published:** 2015-03-26

**Authors:** Michele Tagliabue, Anna Lisa Ciancio, Thomas Brochier, Selim Eskiizmirliler, Marc A. Maier

**Affiliations:** ^1^Neuroscience Research Federation FR3636, CNRS, Université Paris DescartesParis, France; ^2^Centre de Neurophysique, Physiologie et Pathologie, UMR 8119, CNRS, Université Paris DescartesSorbonne Paris Cité, Paris, France; ^3^Laboratory of Biomedical Robotic and Biomicrosystem, Università Campus Bio-Medico di RomaRoma, Italy; ^4^Institut de Neurosciences de la Timone, UMR 7289, CNRS, Aix-Marseille UniversitéMarseille, France; ^5^Life Sciences Department, Université Paris DiderotSorbonne Paris Cité, Paris, France

**Keywords:** precision grip, movement kinematics, EMG, kinematic synergy, muscle synergy

## Abstract

The large number of mechanical degrees of freedom of the hand is not fully exploited during actual movements such as grasping. Usually, angular movements in various joints tend to be coupled, and EMG activities in different hand muscles tend to be correlated. The occurrence of covariation in the former was termed kinematic synergies, in the latter muscle synergies. This study addresses two questions: (i) Whether kinematic and muscle synergies can simultaneously accommodate for kinematic and kinetic constraints. (ii) If so, whether there is an interrelation between kinematic and muscle synergies. We used a reach-grasp-and-pull paradigm and recorded the hand kinematics as well as eight surface EMGs. Subjects had to either perform a precision grip or side grip and had to modify their grip force in order to displace an object against a low or high load. The analysis was subdivided into three epochs: reach, grasp-and-pull, and static hold. Principal component analysis (PCA, temporal or static) was performed separately for all three epochs, in the kinematic and in the EMG domain. PCA revealed that (i) Kinematic- and muscle-synergies can simultaneously accommodate kinematic (grip type) and kinetic task constraints (load condition). (ii) Upcoming grip and load conditions of the grasp are represented in kinematic- and muscle-synergies already during reach. Phase plane plots of the principal muscle-synergy against the principal kinematic synergy revealed (iii) that the muscle-synergy is linked (correlated, and in phase advance) to the kinematic synergy during reach and during grasp-and-pull. Furthermore (iv), pair-wise correlations of EMGs during hold suggest that muscle-synergies are (in part) implemented by coactivation of muscles through common input. Together, these results suggest that kinematic synergies have (at least in part) their origin not just in muscular activation, but in synergistic muscle activation. In short: kinematic synergies may result from muscle synergies.

## Introduction

The human hand with its fingers represents a motion system with many mechanical degrees of freedom (23 DoFs, wrist included), yet it has been observed that simultaneous movements of its joints are rarely independent. Most joint movements show some degree of coupling. Anatomical and neural factors combine to form coordinated joint movements, often referred to as *kinematic synergies*, i.e., simultaneous covariations in (relatively) independent mechanical DoFs (review: Santello et al., 2013). These occur during everyday reach-to-grasp movements of the hand, whether in static postural situations (Santello et al., 1998; Touvet et al., 2014) or during movement (Santello and Soechting, 1998). The presence of kinematic synergies has also been reported during manual exploration (Thakur et al., 2008) or during skilled movements, such as typing (Soechting and Flanders, 1997). Furthermore, *kinetic synergies*, i.e., covariation of forces, have also been observed when generating multi-finger forces, such as in grasp (Santello and Soechting, 2000), between fingers (Grinyagin et al., 2005; Shim et al., 2005) or during handwriting (Hooke et al., 2012).

The existence of kinematic and kinetic synergies raised the question of their origin: the assumption of an underlying covariation of muscle activation (measured by recordings of the electromyogram, EMG) was a straightforward hypothesis. Indeed, spatial and temporal coordination of multiple EMGs, referred to as *muscle synergies*, have been repeatedly observed. Covariation of multiple EMGs during static hand postures (Weiss and Flanders, 2004; Castellini and van der Smagt, 2013) and during active force production was reported: in the latter case synergies between muscles acting on one digit (Valero-Cuevas, 2000), on two (Maier and Hepp-Reymond, 1995b) or more digits (Poston et al., 2010) were found. Muscle synergies have also been found in the non-human primate during grasping (Brochier et al., 2004; Overduin et al., 2008). It is thought that these muscle synergies arise through divergent inputs from premotor neurons to multiple motoneuron pools (Santello et al., 2013).

These observations have led to the hypothesis that the central nervous system (CNS) may not directly control the kinematics (joint angles), nor the kinetics (torques) of the hand, but their underlying synergies. The advantage of that scheme is that synergies typically represent a lower-dimensional space than that formed by the mechanical DoFs of the hand, which should simplify the control problem of the CNS. Similar reasoning would suggest that the CNS controls muscle synergies rather than each muscle independently. In addition, the control of synergies would provide a solution to the problem of apparent kinematic and muscular redundancy (Bernstein, 1967).

The present study explored three issues within the domain of reach-and-grasp synergies: First, we examined whether synergies can accommodate kinematic and kinetic variables conjointly. Usually, the representation of kinematic and kinetic parameters in synergies has been studied separately. Kinetic aspects on their own (kinetic synergies) have usually been taken into account in tasks other than prehension (e.g., Shim et al., 2005; Hooke et al., 2012). Other studies have examined kinetic variables and shown that finger forces (Valero-Cuevas, 2000) or grip forces (Poston et al., 2010) are also represented in low-dimensional EMG space. However, few previous studies have investigated whether muscle synergies can reflect task constraints that combine grasp posture and grasp force. One study looked at upper arm (elbow, shoulder) EMGs relative to isometric force production at the hand under different arm postures (Roh et al., 2012): they found that four muscle synergies could account for the EMG patterns under these task conditions. Here we investigated whether thumb and index finger synergies would account for two task-dependent variables during upper limb prehension: (i) for grip type (precision grip vs. side grip, with clearly different postural kinematics) used to grasp an object and (ii) for load force (low vs. high grip force) in order to hold the grasped object against different load forces. We would expect this to be the case under conditions where the combined control of these two parameters is a necessary precondition for completion of the reach and grasp task.

Second, we compared synergies over time, i.e., over three critical behavioral epochs that constitute our prehension task: the reach period, the grasp-and-pull period and the (static) hold phase. The rational for this is that prehension has been differentiated into reach and grasp, on the conceptual level of motor control (Jeannerod, 1988), on the level of movement kinematics and dynamics (Paulignan et al., 1991; Johansson, 1998), as well as on the level of neural processing (reviews: Castiello, 2005; Grafton, 2010). Given this functional differentiation, kinematic and kinetic constraints may affect synergies differently across these three epochs. Since each component is highly coordinated (Grafton, 2010), we would, however, expect coordination within its respective synergy spaces.

Third, we explored the functional linkage between thumb and index finger kinematic synergies and the corresponding muscle synergies. Few studies have so far attempted a comparison between kinematic and muscle synergies (for static hand postures: Weiss and Flanders, 2004; for static grip: Castellini and van der Smagt, 2013). Establishing such a link is not straightforward: Weiss and Flanders (2004) used multiple regression to map one space into the other, whereas Castellini and van der Smagt (2013) used a distance matrix to compare clustering in the two PC spaces. We put forward a novel concept, namely that muscle synergies may act on and produce kinematic synergies. We provide evidence consistent with this hypothesis.

## Materials and methods

Ten healthy subjects aged between 22 and 37 years (6 females, 4 males, mean age = 29 ± 4 years, all right handed) participated in the behavioral experiment. Subjects gave informed consent, and the procedures, in accordance with the Declaration of Helsinki, were approved by the Institutional Review Board of the Université Paris Descartes (IRB# 00001072).

### Experimental task

**Figure 1:**
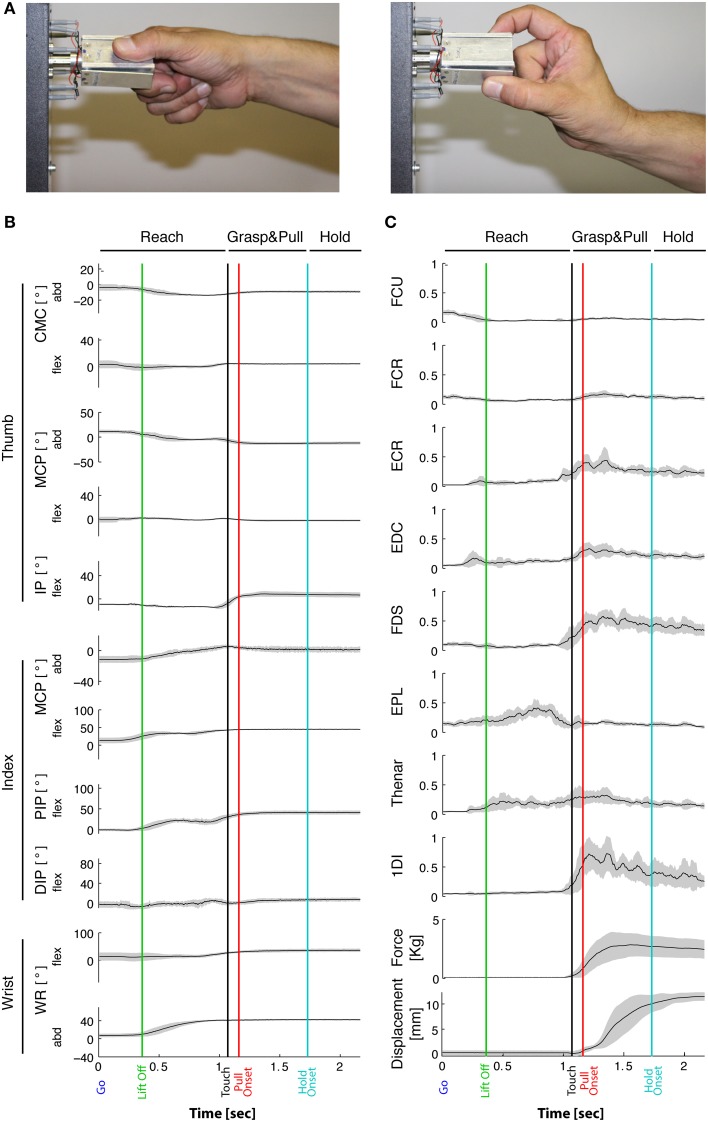
**Task-related movement kinematics and EMGs. (A)** Typical side and precision grip configuration. Grip force is produced between the tip of the thumb and the lateral surface of the index finger in side grip (left), whereas in precision grip (right) force is produced between the tip of the thumb and of the index finger. **(B)** Joint angles over time from a representative subject during the side grip/high force condition. Averaged traces of 12 trials from the Go signal (time 0) to end-hold. Gray-shaded area: ±1 SD. From top to bottom: 5 DoFs of the thumb, 4 DoFs of the index and 2 DoFs of the wrist. Vertical lines: “Lift off” indicates reach onset (green); “Touch onset”: time of object contact (black); Pull onset: onset of object displacement (red); Hold onset: end of displacement and begin of static hold (blue). Horizontal lines on top indicate the *Reach* phase, the *Grasp*&*pull* phase, and the *Hold* period. Positive angular values indicate flexion in the flexion/extension plane, and indicate radial abduction in the adduction/abduction plane. Note little or no qualitative variation in joint angles during *Hold*. **(C)** Corresponding EMGs (same subject and session) normalized to MVC. Two bottom traces: corresponding grip force (thumb and index force summed, in Kg) and horizontal displacement (in mm) of the object. Gray-shaded area: ±1 SD.

The task consisted of a visually guided reach, grasp, pull and hold task. Two task-variables were manipulated: grip type (precision vs. side grip) and pulling load (low vs. high load). Precision grip and side grip involved the thumb and index finger in different spatial configurations and required different postural configurations of the hand (Figure 1A). Subjects were seated in front of a grip manipulandum (partial replica of that in Zaepffel and Brochier, 2012) which consisted of an instrumented grip handle (a parallelepiped of 60 × 38 × 30 mm), rotated 45° from the vertical around its horizontal long axis. Subjects applied grip force on thin stainless steel plates (1 mm thickness, not strictly isometric <2N) equipped with force sensitive resistances (FSR) sensors. Subjects started their hand movement from an initial home position, equipped with a lift-off sensor. Visual cues provided information about the required grip type and load force. Subjects were instructed to reach as soon as this information was available, to grasp the handle according to instruction, and pull the handle horizontally for 12 mm against a load (of either 200 or 700 g), and hold it stationary against the load for 0.5 s. If the subject made a displacement error > 5 mm, the trial was aborted. Similar movement durations among trials were guaranteed by using several constraints, all of which had to be fulfilled for a correct trial: the subject had to start the reach within 1 s after the “go” signal; the handle had to be reached within 2 s; after touching the handle, the subject had to pull it within 1 s; finally, after displacement, the handle had to be hold stable for 0.5 s. For each of the four experimental conditions (precision grip/low load; precision grip/high load; side grip/low load; side grip/high load) 12 trials were completed in a pseudo-randomized order.

### Data acquisition and processing

#### Behavioral data

Custom-written software in Labview 8.5 (National Instruments) was used to control the experimental task and also provided behavioral data on the timing, on the handle displacement, as well as on grip force and load force (forces measured by FSR sensors, sampled at 1 kHz).

#### Hand motion

In order to obtain kinematic data of the thumb, the index finger and the wrist, 17 marker positions were recorded at 200 Hz using the CODA motion analysis system (Charnwood Dynamics LTD, UK). Markers were positioned on the hand according to a previously established protocol (Cordella et al., 2014). Four markers were placed on the index finger _(IF)_: on the metacarpophalangeal (MCP_IF_) joint, on the proximal (PIP_IF_) and distal interphalangeal (DIP_IF_) joints, and on the fingernail. Five markers were positioned on the thumb _(Th)_: two on the carpometacarpal (CMC_Th_), one on the metacarpophalangeal (MCP_Th_) and one on the interphalangeal (IP_Th_) joints, and one on the fingernail. One marker was placed on the MCP joint of the middle finger. Three markers were located on the hand dorsum, one on the wrist, and another three on the arm: on the humeral lateral epicondyles, on the acromion and on sternum respectively.

#### Electromygraphic recording

Surface EMG was recorded at 1 kHz with the Trigno™ wireless EMG system (Delsys, Inc., Boston, MA) from the following eight muscles: Flexor carpi radialis (FCR), Flexor carpi ulnaris (FCU), Extensor carpi radialis (ECR), Flexor digitorum superficialis (FDS), Extensor digitorum communis (EDC), First dorsal interosseus (1DI), Extensor pollicis longus (EPL) and the Thenar muscle group (ThM). Electrode placement followed the guidelines in Criswell (2011). EMGs were smoothed and filtered using a root-mean-square (RMS) algorithm with a 50 ms time-window and a high-pass filter at 30 Hz (Damm and McIntyre, 2008). For statistical analysis, EMGs were normalized to activity at maximal voluntary contraction (MVC). In order to perform PCA analyses of the RMS profiles, EMGs were low-pass filtered at 30 Hz and down-sampled to 100 Hz. For pair-wise correlation of EMGs, but not for PCA, normalization of the EMG was obtained by subtracting the respective average from each EMG (for each muscle and each subject).

#### Trial alignment

For each recorded trial 5 key instants were identified: the Go signal (visual instruction to start the movement); the Touch onset (start of grip force increase on the handle); the Pull onset (start of handle displacement); the Hold onset (beginning of the hold phase: velocity of displacement close to zero, i.e., <10% of peak velocity); the Stop signal (visual instruction to release the handle).

The movement was divided into three phases: *Reach*, *Grasp*&*pull* and *Hold* (Figure 1C), which were defined as follows:

- *Reach*: from Go to Touch onset.
- *Grasp*&*pull*: from Touch onset to Hold onset, which corresponds to the grasp and displacement of the handle.
- *Hold*: from Hold onset to Stop, which corresponds to the static holding of the handle against the load.

All behavioral, kinematic and EMG data were normalized in time and resampled to the grand average duration for *Reach* and for *Grasp*&*pull*. In contrast to these two “dynamic” periods, the *Hold* phase was considered static, and the time-averaged values of the dependent variables in this phase were used for analyses.

#### EMG cross-talk

To assess potential artifactual cross-talk between EMGs, cross-correlations of any given pair of EMGs within a subject were computed based on the raw EMG signals of all trials. Cross-talk was assumed to be present for a correlation coefficient of *r* > 0.3 at lag = 0 (Brochier et al., 2004) and *p* < 0.0018 using a Bonferoni correction, see below. Out of the 280 pairs tested, four showed cross-talk according to these criteria: FCR-FCU (in two subjects), ECR-FCR and EPL-FDS (each in one subject only). Subsequently, 1 EMG from these four muscle-pairs (a total of 28 pair-wise comparisons) was deleted from the correlational analysis on pair-wise muscle synergies. However, all subjects and EMGs were maintained for the PCA since this method seeks to decorrelate potentially correlated input: PCA does not provide false positive results in case of cross-talk in the input. We therefore judged elimination of four subjects, or elimination of two EMGs in all subjects, as more detrimental than presence of some cross-talk in the PCA.

### Data analysis

#### Movement kinematics

The hand configuration (joint angles of the thumb, index finger and wrist, a total of 11 DoFs, Figure 1B) was reconstructed from the marker readings (Cordella et al., 2014). The markers on the index finger provided the following four DoFs: flexion/extension of MCP_IF_, PIP_IF_, and DIP_IF_, and abduction/adduction of MCP_IF_. The following five thumb DoFs were extracted: flexion/extension of CMC_Th_, MCP_Th_,IP_Th_, and abduction/adduction of CMC_Th_ and MCP_Th_. Two further DoFs were extracted from the markers on the hand dorsum: wrist flexion/extension (WR_flex_) and wrist radial/ulnar abduction (WR_abd_).

#### Statistical analysis

Shapiro-Wilk test for normality of the dependent variables was performed prior to ANOVA: joint angles and EMG during hold were normally distributed (*p* < 0.05 for each joint angle, *p* < 0.05 for each EMG).

Repeated measures ANOVA was used to assess the influence of the independent variables GRIP (precision grip/side grip) and LOAD (low/high load) on the dependent variables: either on the 11 DoFs of the joint angles, or on the eight EMGs. This was done during *Hold*. For the sake of clarity, we refer to the independent factors GRIP and LOAD of the ANOVA in capital letters throughout the article.

#### Principal component analysis (PCA)

In order to assess the kinematic synergies, a separate PCA was applied to each behavioral epoch: (i) to the time-varying kinematics during *Reach*, (ii) to the time-varying kinematics during *Grasp*&*pull*, and (iii) to the static kinematic configuration during *Hold*. Trials were excluded if visual occlusions did not allow the reconstruction of all 11 joint profiles (see Table 1).

**Table 1:** **PCA matrix size (rows × columns) for PCA on the kinematics and for PCA on the EMGs**.

	**Reach**	**Grasp&pull**	**Hold**
**PCA ON kINEMATICS**			
Theory	29 bins × 5280 var.^*^	16 bins × 5280 var.	480 var. × 11 joint angles
Artifact removal	29 bins × 5056 var. (−4%)	16 bins × 5171 var. (−2%)	459 var. (−4%) × 11
**PCA on EMGs**			
Theory	29 bins × 3840 var.	16 bins × 3840 var.	480 var. × 8 EMGs
Artifact removal	29 bins × 3760 var. (−2%)	16 bins × 3760 var. (−2%)	470 var. (−2%) × 8 EMGs

*Bins, number of sampled bins for time-varying joint angles or EMGs; var, variables*.

For the static *Hold* period, a conventional PCA was performed on the time-average of the joint angles. The dataset used for this analysis, $\vec{\phi }$, consisted of the ensemble of all trials performed by all subjects, where each trial was represented by an 11-dimensional vector (see Table 1 for PCA matrix dimensions). The PCA provides an orthogonal system of eigenvectors (principal components) in the joint space along which the data are preferentially distributed. We retained PCs that each explained >5% of the variance, and which together and in decreasing order explained >85% of the total variance. Each eigenvector can be seen as a “synergy,” representing the covariation, a linear combination, of the joint angular configurations. The contribution of each joint (its variation) to every PC is expressed by its *loading*. The *PC scores* associated to a given trial (posture) correspond to the location of this trial within the PC space (Castellini and van der Smagt, 2013). It follows that the modulation between trials of the scores associated to the *k*th eigenvector represents a movement of the hand configuration along the *k*th synergy. For statistically assessing the impact of the independent variables on the joint configuration represented in the PC space, a two-way (GRIP, LOAD) repeated measures ANOVA was performed on the PC-scores of each retained PC.

For the *Reach* and *Grasp* & *pull* epochs, a temporal PCA was performed on the dataset $\vec{\theta }$(*t*), consisting of the ensemble of all joint angle time-profiles for all trials and subjects (Thomas et al., 2005). This procedure allows computing of the *temporal weighting*, *PC_k_*(*t*), of the different PCs, i.e., the projection of $\vec{\theta }$(*t*) on the different eigenvectors $\vec{v_{k}}$ obtained by the PCA (Daffertshofer et al., 2004). Since *PC_k_*(*t*) = *v_k_* · $\vec{\theta }$(*t*), then the *n*th component of an eigenvector $\vec{v_{k}}$ can be seen as the coefficients associating the *n*th time profile of the original data set to *PC_k_*(*t*), i.e., it expresses the gain with which a given joint angular profile acts on the temporal weighting identified by the PCA. For statistically assessing the impact of the independent variables on the joint time-profile, a three-way (GRIP, LOAD, JOINT) repeated measures ANOVA was performed on the PC coefficients of each retained PC.

Similarly for the EMGs: a PCA was performed for each of the three behavioral epochs. For *Reach* and *Grasp*&*pull* on the time-varying EMGs, for *Hold* on the time-average during the hold period. Trials with EMG artifacts were removed from PCA analysis. Table 1 shows the theoretical matrix dimensions and the used dimensions after artifact removal. There are three theoretical corner values:

- Number of experimental conditions: 480 = 10 subjects * 12 trials * 2 grip types * 2 load forces.
- For kinematics: 5280 = (A) * 11 joint angles.
- For EMGs: 3840 = (A) * 8 EMGs.

Furthermore, to compare PCs in the kinematic domain to those of the EMG domain, phase plane plots of their temporal weighting profiles *pc*_k_(*t*) were computed (over identical time periods).

#### Pair-wise muscle synergies

Pair-wise covariation of EMG activity was assessed subject by subject. In contrast to PCA of EMG, which captures the covariance of (time-varying or static) EMG amplitude as a function of the independent variables, pair-wise correlation examines the trial-by-trial covariation around the mean activity, since the average of the EMG activity per condition was subtracted from each corresponding trial. This eliminates grip type and load force as confounding variables. Relative EMG amplitude during *Hold* was determined trial-by-trial and Pearson correlations between two EMGs calculated. Correlations were classified into “co-activation” (*r* > 0, *p* < 0.0018), “reciprocal activation” (*r* < 0, *p* < 0.0018) or absent (*p* > 0.0018), applying a Bonferoni correction (*N* = 28 of the lower 8 × 8 triangular matrix of EMGs, **Figure 8**) for a single-test equivalent of *p* = 0.05.

## Results

Typical behavioral, kinematic, and EMG signals are depicted in Figure 1 for a single subject. Analysis was split into the three successive periods of *Reach*, *Grasp*&*pull*, and *Hold* (Figures 1B,C). Note that grip force on the handle increased till it was sufficient to horizontally displace and then hold the handle against the load (Figure 1C). Grip force increased during *Grasp*&*pull* and then remained constant during *Hold*.

### Task-related movement kinematics

#### Joint configuration during reach and grasp&pull

The task-related reach-grasp-and-hold kinematics of the index finger, thumb, and wrist are shown in Figure 1B for a representative subject in the side grip/high load condition. During the movement phase of the reach, i.e., between movement onset (green vertical line) and object contact (black vertical line), the hand underwent preshaping primarily through thumb adduction, index finger flexion, and wrist abduction. Across subjects, movement duration (including reaction time) of the reach was on average 1.4 ± 0.2 s. It did vary as a function of load [faster for higher loads, *F*_(1, 9)_ = 18.24, *p* = 0.002], but not as a function of grip type [*F*_(1, 9)_ = 2.47, *p* = 0.15]. Reach was followed by object contact and grip formation. In general, this was achieved essentially through adduction (MCP_Th_) and flexion (IP_Th_) of the thumb, and flexion (PIP_IF_, DIP_IF_) and radial abduction (MCP_IF abd_) of the index finger (as shown in Figure 1B). For certain joints, such as MCP_Th_, PIP_IF_ and DIP_IF_, preshaping and grip formation marked two clearly distinct periods, whereas other joints showed a more monotonous variation, such as index finger abduction (MCP_IFabd_). Therefore, the time profile of thumb and index finger kinematics was not uniform.

#### Joint configuration during static hold

**Figure 2:**
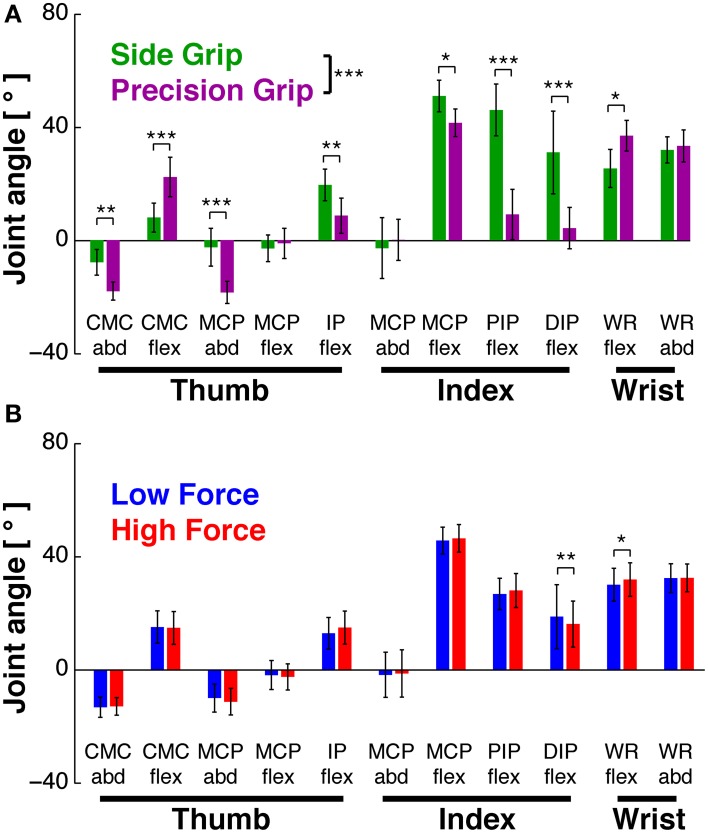
**Hand posture as a function of task conditions**. Joint angles during *Hold*, averaged across subjects (mean±95% confidence interval). **(A)** Joint angles in side (green) vs. precision grip (purple), independent of load condition. GRIP showed a significant main effect (^***^at legend): 8 of 11 joints showed significant differences in *post-hoc* tests. **(B)** Joint angles in low (blue) vs. high load condition (red), independent of grip type. There was no significant main effect of LOAD (but an interaction effect, in which two joints showed a significant difference in *post-hoc* tests). ^*^At individual joints: significant difference of *post-hoc* test at *p* < 0.05, ^**^p < 0.01, ^***^p < 0.001.

ANOVA showed that GRIP [*F*_(1, 9)_ = 35.1, *p* < 0.001], but not LOAD [*F*_(1, 9)_ = 0.5, *p* > 0.05] had a significant main effect on the grip configuration. There was also a significant interaction between JOINT^*^GRIP [*F*_(1, 9)_ = 18.1, *p* < 0.001] and between JOINT^*^LOAD [*F*_(1, 9)_ = 3.2, *p* < 0.001]. This, and the results of *post-hoc* tests are illustrated in Figure 2. As expected, all seven joints (i.e., 8 out of 11 DoFs) showed a significant variation with GRIP (Figure 2A): for the index finger all three joints were more flexed in side grip (*p* = 0.02, *p* = 0.0002, *p* = 0.0002), whereas abduction did not vary (*p* = 0.84). For the thumb, the IP joint was also more flexed in side grip (*p* = 0.009), but not the MCP joint (*p* = 0.95), which was, however, less abducted (*p* = 0.0004). The CMC joint was less flexed (*p* = 0.0001), but more abducted in side grip (*p* = 0.006). Wrist flexion was stronger in precision grip (*p* = 0.01), but its abduction did not vary (*p* = 0.69). The quantitative effect of LOAD is shown in Figure 2B: among the seven joints, two (i.e., 2 out of 11 DoFs) varied as a function of load force: the distal index finger joint (DIP_flex_, *p* = 0.005) and the wrist (WR_flex_, *p* = 0.05): the former was less, the latter more flexed during high load. This was more surprising, since each of the two static grip configurations was, on a qualitative level, considered isometric, i.e., invariant with respect to grip or load force.

### Task-related EMGs

#### EMGs during reach and grasp&pull (qualitative comparison)

The task-related EMGs of a representative subject (same as in Figure 1B) are shown in Figure 1C. Muscles acting on the wrist, such as FCU, FCR and ECR, were deactivated prior to movement onset and were not or weakly active during reach. However, they were reactivated during grip formation and during hold (ex. ECR). Functionally, this probably represents stabilization of the wrist in order to apply grip and pull forces. Thumb and index finger muscles implied in grip force production (agonists such as FDS, 1DI, and Thenar muscles) increased their activity during grip formation and maintained (or slowly decreased) their activity during hold. Furthermore, antagonists were either co-contracted during grip formation and hold (such as EDC), or implicated during preshaping (ex. EPL). Thus, as was the case in kinematics, the EMGs also showed non-uniform time-varying profiles.

#### EMGs during static hold

**Figure 3:**
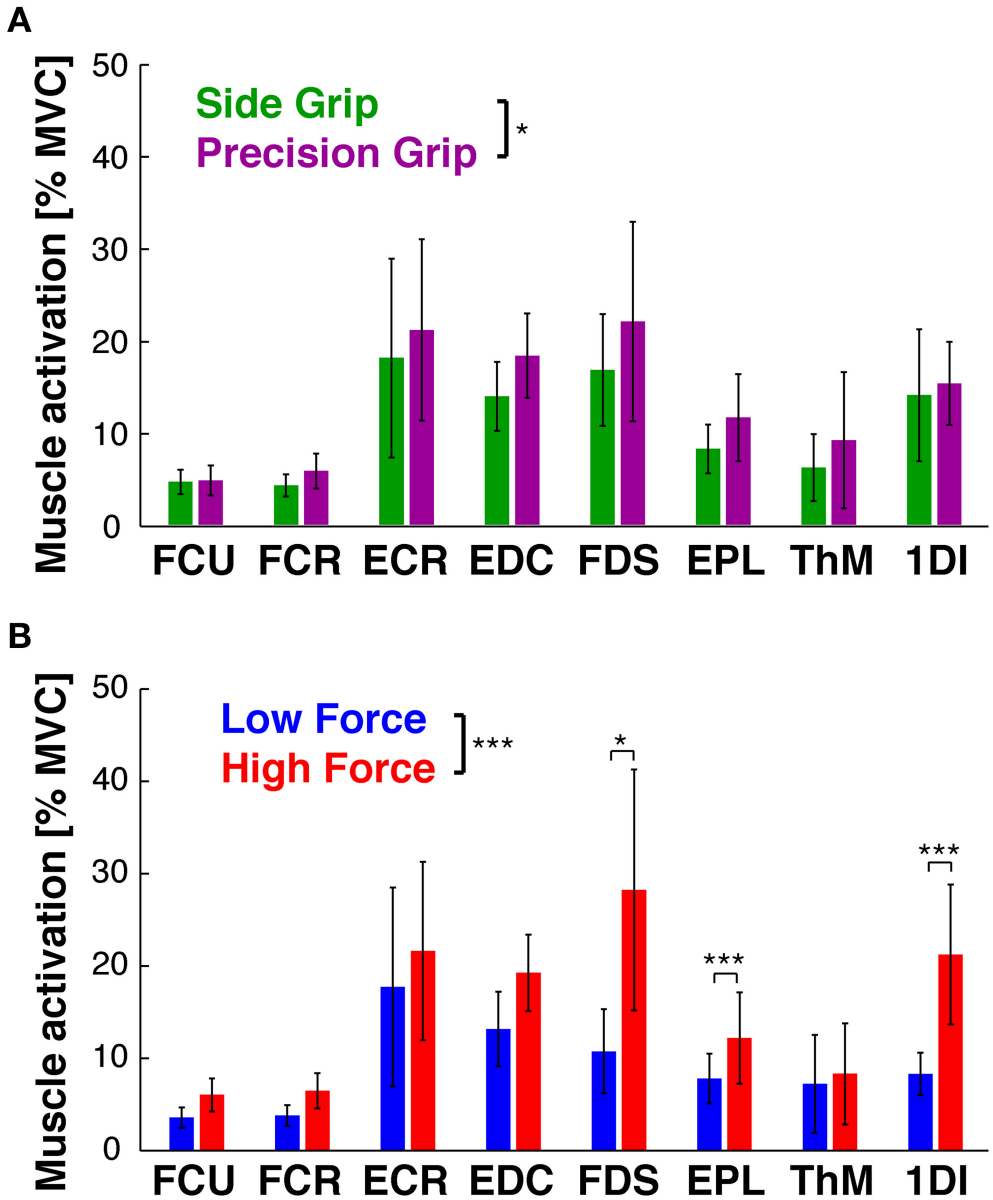
**EMG as a function of task conditions**. EMG during *Hold*, averaged across subjects (mean ± 95% confidence interval). **(A)** EMGs in side (green) vs. precision grip (purple), independent of load condition. GRIP showed a significant main effect (^*^at legend): precision grip usually evoked higher EMG activity. **(B)** EMGs in low (blue) vs. high force condition (red), independent of grip type. LOAD showed a significant main effect (^***^ at legend), with FDS, EPL and 1DI showing significantly increased activity in *post-hoc* tests. ^*^At individual EMGs: significant difference of *post-hoc* test at *p* < 0.05, ^**^p < 0.01, ^***^p < 0.001.

EMGs were modulated as a function of grip type (Figure 3A) and load force (Figure 3B). ANOVA showed significant main effects of GRIP [*F*_(1, 9)_ = 6.9, *p* < 0.05] and of LOAD [*F*_(1, 9)_ = 26.5, *p* < 0.001). Most muscles increased their activity with the higher load, some of them significantly (Figure 3B): the force producing agonists FDS (*p* = 0.02) and 1DI (*p* = 0.0001) as well as the antagonist EPL (*p* = 0.0001).

### Grip posture during hold: PCA of kinematics and of EMG

#### Kinematics

**Figure 4:**
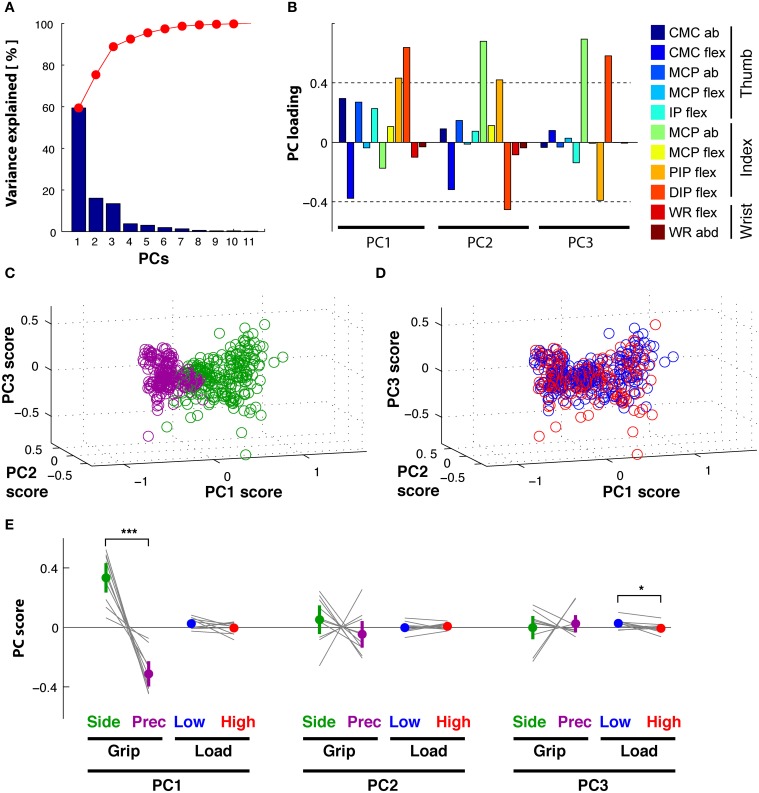
**Hold period: PCA on kinematics. (A)** Scree plot: percentage of explained variance in the kinematics by each PC (blue bar, Eigenvalue in %) and cumulative percentage explained (red). **(B)** Loadings of each DoF (color-coded) per PC. **(C)** PC space formed by the first three PCs: each trial (circle) is represented by its PC scores. PC1 visibly separates side grip trials (green) from precision grip trials (purple). **(D)** PC space of the first three PCs: no visible separation of low (blue) vs. high (red) load force trials in the PC scores (but see **E**). **(E)** Average PC scores (colored dot) and ±95% confidence interval (colored vertical bar) for each PC and task condition (thin gray lines correspond to the average scores for each subject). Significant difference in average PC scores on PC1 for grip type and in PC3 for load condition (note: this latter difference is, however, not visible in the PC space **D**).

Our previous statistical analysis during the hold period showed that 8 out of 11 DoFs varied as a function of grip type, and that few DoFs varied with the two load conditions. A PCA on these static joint angles showed, however, that this manifold (or 89% of its variance) could be reduced to three PCs (Figure 4A): PC1 explained 60%, PC2 16%, and PC3 13%. The remaining 6 PCs explained between 4 and 0.2% each and were not retained. Figure 4B shows the contribution (loading) of each joint angle to the first three PCs. Clearly, distal index finger flexion (PIP_IF_ and DIP_IF_) dominated (loading > 0.4 or < -0.4) in PC1, whereas index finger abduction (MCP_IF_) and flexion dominated (PIP_IF_, DIP_IF_) in PC2 and PC3. The DoFs of the thumb were most present in PC1, less so in PC2 and virtually absent in PC3. The two wrist angles were only marginally represented. Interestingly, these three PCs differentiated the independent variables: PC1 separated the trials into precision and side grip. This is evident on visual inspection of Figure 4C, a graphical representation of the PC scores associated to the first three eigen vectors of the PC-space (precision grip trials: purple; side grip: green). Grip-type separation on PC1-scores was confirmed by ANOVA [GRIP, *F*_(1, 9)_ = 57.0, *p* < 0.001]. Although not visible in the PC-space in Figure 4D, PC3 differentiated low from high load trials, as confirmed by ANOVA [LOAD, *F*_(1, 9)_ = 7.6, *p* < 0.05]. The significant differences in PC scores are more clearly shown in Figure 4E, which depicts the average score (colored dot) for each condition and that of each subject (thin gray lines). All corresponding statistical results are given in Table 2.

**Table 2:**
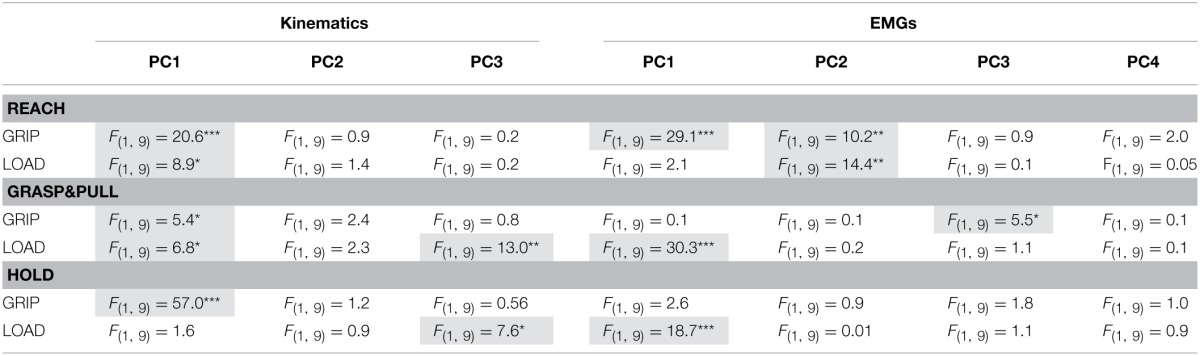
**Relation between PC coefficients (or PC scores in the static hold period) and the two independent variables (GRIP and LOAD) according to ANOVA for each PC and behavioral epoch**.

Shown are F_(treatment factor df, within group df)_-values, ^*^p < 0.05, ^**^p < 0.01, ^***^p < 0.001 from ANOVA on each PC. ANOVA factors are: GRIP (precision vs. side grip) and LOAD (low vs. high load). Only main effects are given. Gray-shaded: significant F-value (p < 0.05).

#### EMG

**Figure 5:**
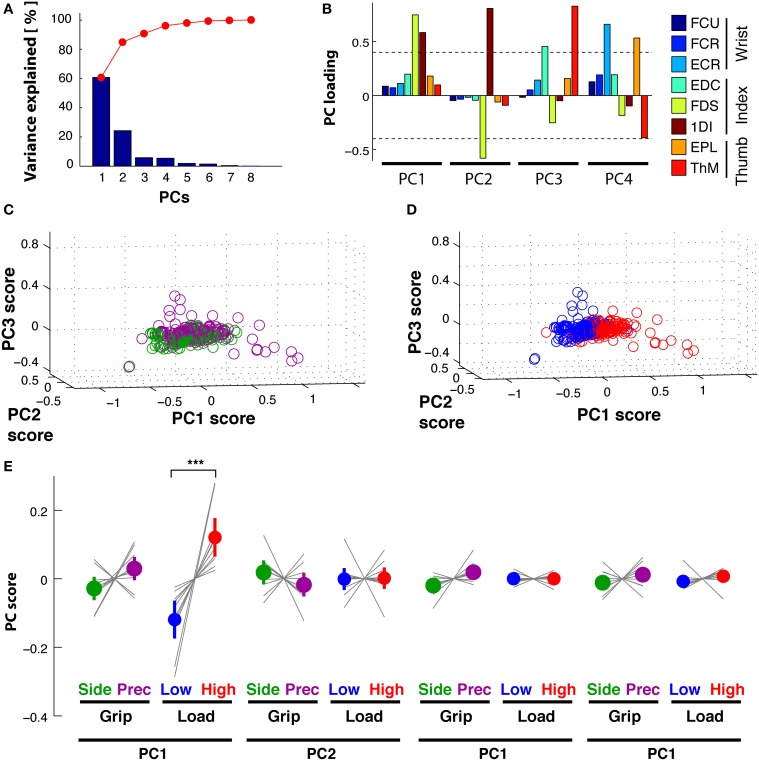
**Hold period: PCA on EMG. (A)** Scree plot: percentage of explained variance in the EMGs by each PC (blue bar, Eigenvalue in %) and cumulative percentage explained (red). **(B)** Loadings for each EMG (color-coded) per PC. **(C)** PC space formed by the first three PCs: each trial is represented by its PC scores. No visible separation between PC scores of side grip (green) vs. precision grip trials (purple). **(D)** PC space of the first three PCs: visible separation between PC scores of low (blue) vs. high (red) load trials on PC1. **(E)** Average PC scores (colored dot) and ±95% confidence interval (colored vertical bar) for each PC and task condition (thin gray lines correspond to the average scores for each subject). Significant difference in average PC scores on PC1 for LOAD (high vs. low).

PCA on the EMGs during static hold resulted in four retained PCs that explained 96% of the total variance (Figure 5A). PC1 explained 61%, PC2 24%, PC3 6%, and PC4 5%. The loading of the eight EMGs to each PC was qualitatively different (Figure 5B): index finger muscles dominated (loading > 0.4 or < −0.4) in PC1 (1DI, FDS) and PC2 (1DI, -FDS), whereas intrinsic thumb muscle (Thenar) and EDC dominated in PC3. PC4 was dominated by thumb muscles (EPL and Thenar) and a wrist muscle (ECR). Flexor wrist muscles (FCU, FCR) were only marginally involved in any of the PCs. In contrast to the PC scores of the kinematics, the EMG PC scores allowed for significant differentiation of load condition only, but not of grip type: the PC-space in Figure 5C shows the overlap of side and precision grip trials, whereas Figure 5D shows the clear differentiation between low and high load trials on PC1 [LOAD, *F*_(1, 9)_ = 18.7, *p* < 0.001]. This is shown again in Figure 5E for the average PC scores and those of each subject (also see Table 2).

These results suggest that the joint and EMG manifold can be reduced, during static *Hold*, to a lower dimensional synergistic space that still differentiates grip type and load force in the kinematic PC space, and load force in the EMG PC space. How these kinematic and EMG patterns evolve over time during *Reach* and during *Grasp*&*pull* in order to form the static pattern at the end of the task will be investigated in the following.

### Reach period, grasp&pull period: time-resolved PCA of kinematics

#### Reach

**Figure 6:**
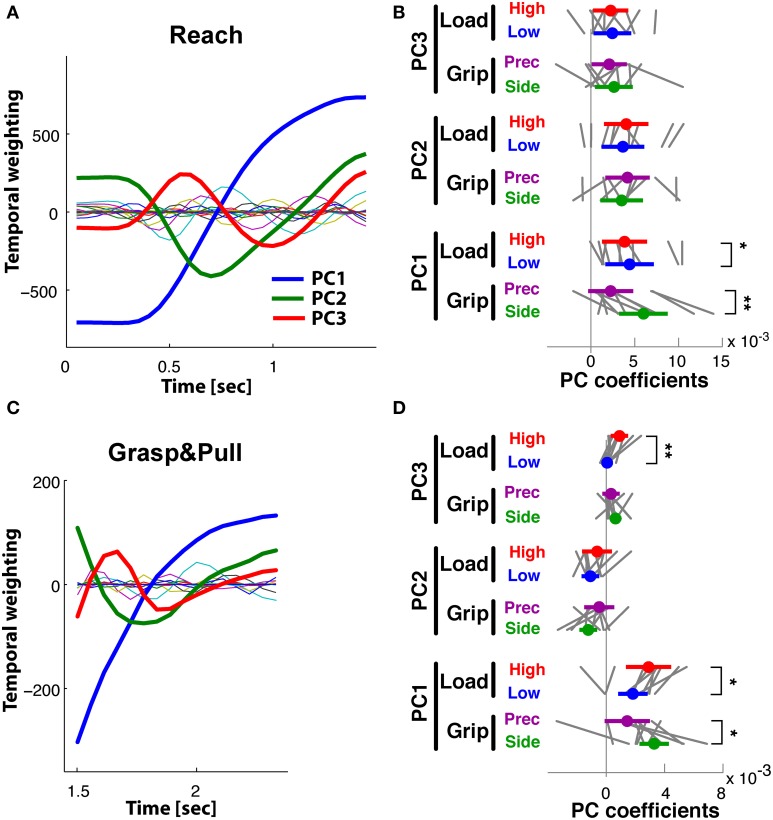
**Reach period compared to grasp&pull period: PCA on time-varying kinematics. (A,B)** *Reach* period. **(C,D)** *Grasp*&*pull* period. (**A)** *Reach*: temporal weighting for the first three PCs (thick lines) and the other PCs. **(B)** *Reach*: Average PC coefficients (colored dot) and ±95% confidence interval (colored horizontal bar) per PC and task condition, and corresponding averages for each subject (thin gray lines). Significant difference in average PC coefficients on PC1 for grip type and load condition. (**C)** *Grasp*&*pull*: temporal weighting for the first three PCs (thick lines) and the other PCs. **(D)** *Grasp*&*pull* (scheme as in **B**): significant difference in average PC coefficients on PC1 for GRIP, and on PC1 and PC3 for LOAD. ^*^, ^**^ Indicate significant difference at *p* < 0.05 and *p* < 0.01, respectively.

PCA of the time-varying kinematics during *Reach* resulted in three PCs explaining 95% of the total variance. PC1 explained 76%, PC2 14% and PC3 5%, while the other not retained PCs explained the remaining 5%. The first three temporal weighting curves (thick lines) are shown with those of the further PCs (thin lines) in Figure 6A: these first three curves showed clearly distinct weighting profiles. *PC_1_*(*t*) (blue) showed a monotonic increasing profile, starting to increase at movement onset and reaching its maximum at object contact. *PC_2_*(*t*) (green) had a non-monotonic single-peaked profile, with a maximal negative value at about the estimated time of maximum aperture. This profile was reminiscent of double peaked angular profiles during reach, such as index finger DIP_IF_ and PIP_IF_ joints (Figure 1B). *PC_3_*(*t*) (red) showed a doubled-peaked profile with a first maximum prior to and a second peak after that of *PC_2_*(*t*). Figure 6B illustrates the distribution of grip- and load-dependent PC coefficients for the average (colored dot and vertical bar) as well as for each subject (thin gray lines). Side grip trials had significantly larger [GRIP, *F*_(1, 9)_ = 20.6, *p* < 0.001) PC coefficients on *PC_1_*(*t*) than precision grip trials (purple), but not for *PC_2_*(*t*) and *PC_3_*(*t*). In addition, PC coefficients associated to *PC_1_*(*t*) also showed a significant (but smaller) difference for low and high load conditions [LOAD, *F*_(1, 9)_ = 8.9, *p* < 0.05, Table 2 for complete ANOVA results).

#### Grasp&pull

A similar analysis was undertaken for the *Grasp*&*pull* period. Again the first three PCs explained 96% of the variance (PC1 explained 78%, PC2 13%, and PC3 5%). Figure 6C shows the temporal weighting profiles: *PC_1_*(*t*) (blue) increased monotonically, *PC_2_*(*t*) (green) was non-monotonic with a single negative peak. *PC_3_*(*t*) (red) was bi-phasic, with an early positive peak and a slower increase toward the end of the pull. Statistical analysis (Table 2) showed a significant effect of GRIP on the PC coefficients of *PC_1_*(*t*) [F_(1, 9)_ = 5.4, *p* < 0.05] and of LOAD on the coefficients of *PC_1_*(*t*) and *PC_3_*(*t*), illustrated in Figure 6D [*F*_(1, 9)_ = 6.8, *p* < 0.05 and *F*_(1, 9)_ = 13.0, *p* < 0.01, respectively].

### Reach period, grasp&pull period: time-resolved PCA of EMG

#### Reach

**Figure 7:**
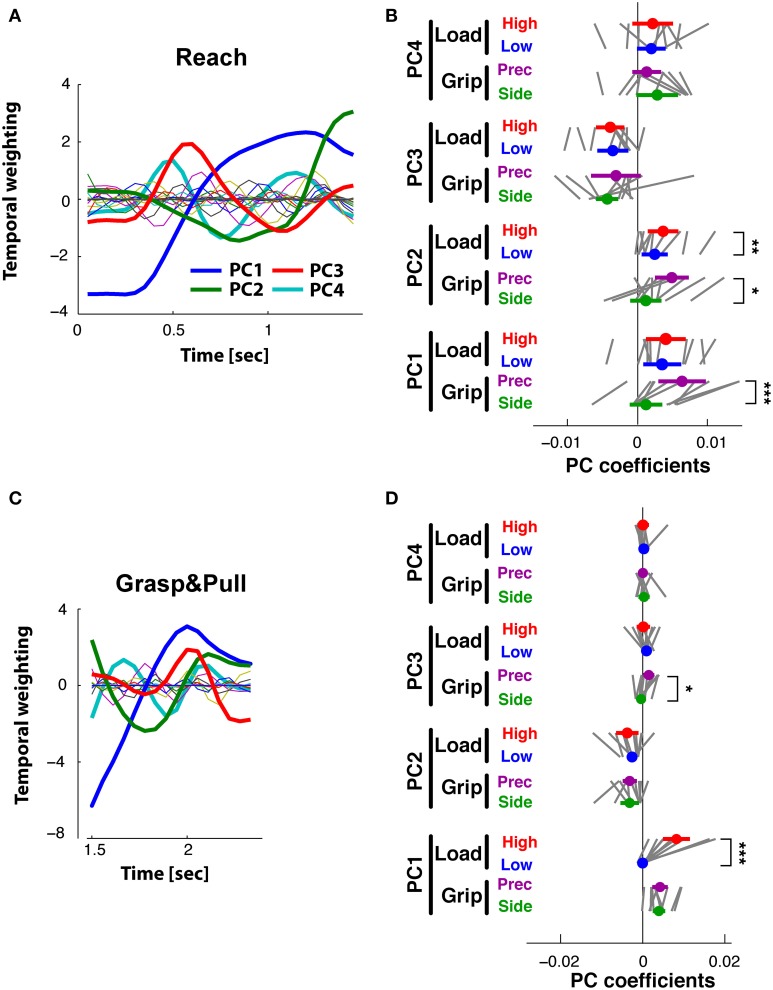
**Reach period compared to grasp&pull period: PCA on time-varying EMGs. (A,B)** *Reach* period. **(C,D)** *Grasp*&*pull* period. **(A)** *Reach*: temporal weighting for the first four PCs (thick lines) and the other PCs. **(B)** *Reach*: Average PC coefficients (colored dot) and ±95% confidence interval (colored horizontal bar) for each PC and task condition (thin black lines correspond to the average coefficients for each subject). Significant difference in average PC coefficients on PC1 and PC2 for GRIP, and on PC2 for low vs. high LOAD. (**C)** *Grasp*&*pull*: temporal weighting for the first four PCs (thick lines) and the other PCs. **(D)** *Grasp*&*pull* (scheme as in **B**): significant difference in average PC coefficients on PC1 for LOAD, and on PC3 for GRIP. ^*^, ^**^, ^***^ Indicate significant difference at *p* < 0.05, *p* < 0.01 and *p* < 0.001, respectively.

PCA of the time-varying EMGs during *Reach* resulted in 4 PCs explaining 88% of the variance. We retained these first four PCs, which explained 55, 18, 9, and 6% of the variance, respectively. The temporal weightings of all PCs are shown in Figure 7A. The profile of *PC_1_*(*t*) (blue line) was single-peaked but non-monotonic with a maximum at about 1.2 s, just prior to object contact, whereas the profile of *PC_2_*(*t*) (green line) had an earlier negative peak followed by a maximum at time of contact. *PC_3_*(*t*) (red line) had an early positive peak (at about 0.6 s) followed by a negative peak (at about 1.1 s). *PC_4_*(*t*) (light blue) had three peaks. The PC coefficients of *PC_1_*(*t*) and *PC_2_*(*t*) visibly differentiated the trials according to grip type (Figure 7B, shown for the average and for each subject). ANOVA confirmed that GRIP significantly affected the *PC_1_*(*t*) and *PC_2_*(*t*) coefficients [*F*_(1, 9)_ = 29.1, *p* < 0.001 and *F*_(1, 9)_ = 10.2, *p* < 0.01, respectively]. In addition those of *PC_2_*(*t*) also differentiated the trials according to LOAD [*F*_(1, 9)_ = 14.4, *p* < 0.01]. Table 2 provides a summary of the significant and non-significant results of the ANOVA.

#### Grasp&pull

PCA of the time-varying EMGs during the *Grasp*&*pull* period resulted in 4 PCs explaining 93% of the variance (61, 17, 9, and 6%, respectively). The temporal weighting of the PCs are shown in Figure 7C: *PC_1_*(*t*) (blue line) was single-peaked but non-monotonic with a maximum at about 2 s, probably at max. grip force, whereas *PC_2_*(*t*) (green line) had an earlier negative peak. *PC_3_*(*t*) (red line) had a positive peak (at about the max. of PC1) followed by a pronounced negative peak at the end of the pull (at 2.5 s). *PC_4_*(*t*) showed a triple-peaked profile. The PC coefficients visibly differentiated the trials according to GRIP on *PC_3_*(*t*) [*F*_(1, 9)_ = 14.4, *p* < 0.01) and according to LOAD on *PC_1_*(*t*) [*F*_(1, 9)_ = 30.3, *p* < 0.01] as shown in Figure 7D for the average and for each subject. No other significant dependencies were found (Table 2).

### Occurrence and types of pair-wise muscle synergies

**Figure 8:**
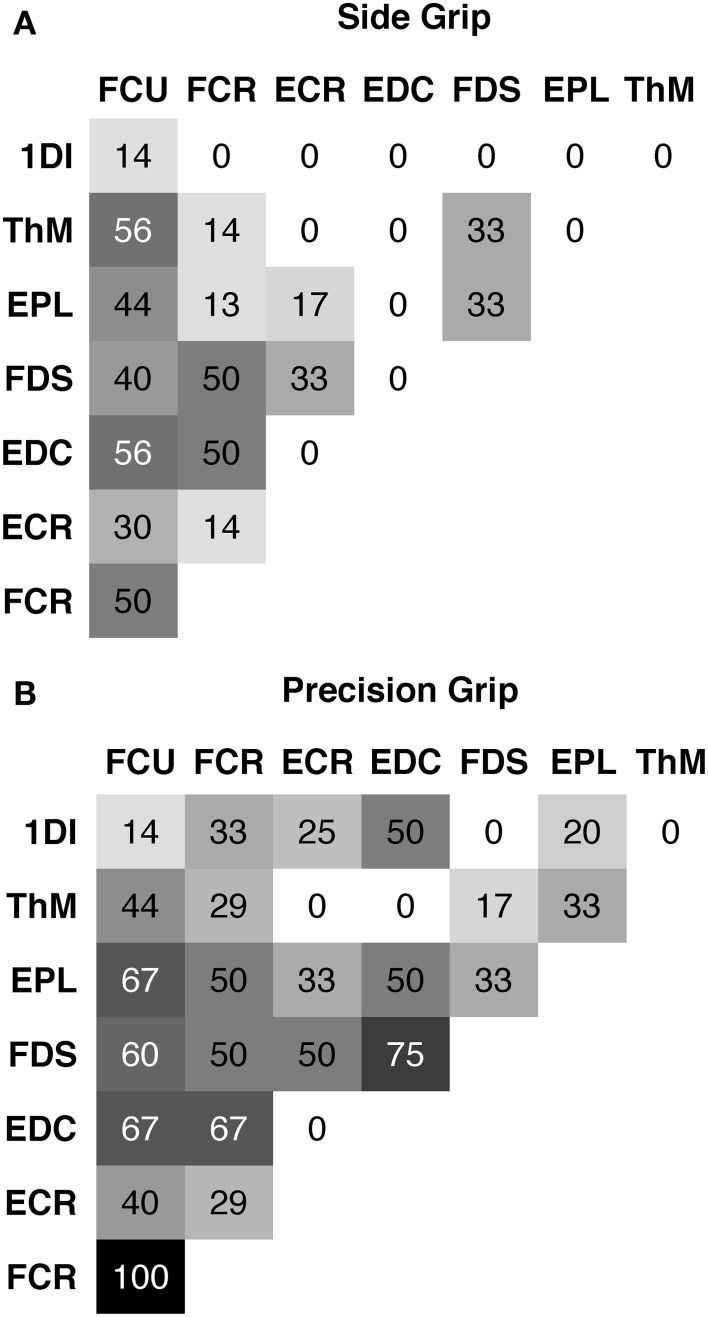
**Pair-wise muscle synergies during hold**. Each matrix shows the occurrence of a significant positive correlation (coactivation) between two EMGs expressed in % of subjects. **(A)** for side grip, **(B)** for precision grip. Note: overall more coactivation in precision grip (grand average = 37%) than side grip (grand average = 20%).

Although the lower-dimensional PCA space indicated the presence of muscular synergies, the PCA did not allow for an investigation of potential underlying mechanisms. This was investigated in a complementary and indirect analysis using correlations between pairs of EMGs. Two types of muscle synergies, i.e., trial-to-trial covariation of EMG, were defined: coactivation and reciprocal activation, represented by a positive and negative correlation, respectively. The triangular matrix of the 28 muscle pairs showing the occurrence of muscle synergies during the hold period is shown in Figure 8. Occurrence is indicated in percentage of subjects with a significant correlation in a particular muscle pair. Side grip (Figure 8A), elicited less coactivation (grand average = 20%) than did precision grip (Figure 8B, 37%). Independent of grip type, coactivation was more frequent between the 15 different pairs of extrinsic muscles (average = 40%), than between the 12 extrinsic–intrinsic muscle pairs (16%). No case of reciprocal activation was found. A similar analysis was undertaken for the amplitude of the peak-EMG activity and its latency over the entire trial: covariation of peak amplitude was generally less frequent, but showed a similar gradient for grip type (19% for side grip, 29% for precision grip). Covariation of peak timing was least frequent (4% for side grip, 8% for precision grip).

### Functional link between EMG and kinematic synergies

**Figure 9:**
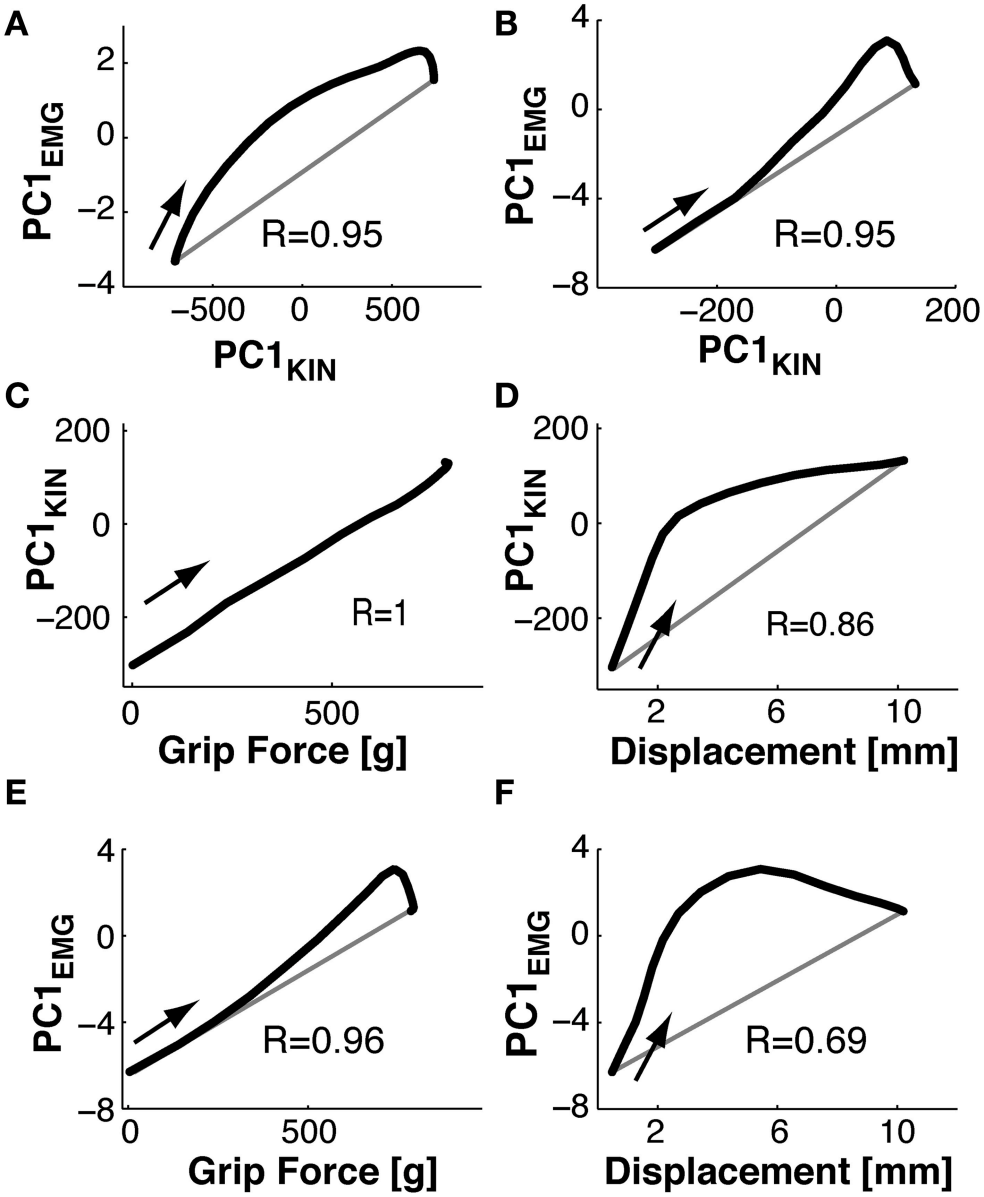
**Phase plane plots. (A,B)** Functional link between muscle and kinematic synergies. **(C,D)** Functional link between kinematic synergies and grip force or displacement during *Grasp*&*pull*. **(E,F)** Functional link between EMG synergies and grip force or displacement during *Grasp*&*pull*. **(A)** During *Reach*: phase plane of the PC1 temporal weighting in the EMG-space against the PC1 temporal weighting in the kinematics-space i.e., *PC_1_*(*t*)_EMG_vs.*PC_1_*(*t*)_KIN_. Note the phase advance of *PC_1_*(*t*)_EMG_ (above the gray diagonal between the start and end point) against*PC_1_*(*t*)_KIN_. **(B)** During *Grasp*&*pull*: phase plane of *PC_1_*(*t*)_EMG_vs.*PC_1_*(*t*)_KIN_. Note progressive phase advance of *PC_1_*(*t*)_EMG_.**(C)** Phase plane *PC_1_*(*t*)_KIN_vs.grip force. Note perfect correlation, no time lag. **(D)** Phase plane *PC_1_*(*t*)_KIN_vs.displacement, showing loose coupling and strong phase advance of *PC_1_*(*t*)_KIN_ with displacement. **(E)** Phase plane *PC_1_*(*t*)_EMG_vs.grip force, with strong correlation and increasing phase advance of *PC_1_*(*t*)_EMG_. **(F)** Phase plane *PC_1_*(*t*)_EMG_vs.displacement, with loos coupling and strong phase advance of *PC_1_*(*t*)_EMG_ with displacement. The arrow in each graph indicates the temporal direction (start) of the phase-plane trajectory.

To compare kinematic to muscle synergies we computed the phase space of the respective PCs, given the underlying hypothesis that the two should be linked since variations in joint angles are (in part) caused by EMG activity. Note that the kinematic and the muscle synergies had been computed fully independently of each other. Figure 9A shows the phase plane plot of PC1_EMG_ against PC1_KIN_ during *Reach*, i.e., the trajectory of their respective temporal weighting. Clearly, the temporal evolution of these two PCs was related, expressed by a parallel and (essentially) monotonous increase over time. However, there was a consistent phase-advance of the PC_EMG_, (of up to 50 ms) indicated by the area above the gray line from the start to the end point. This was also the case during *Grasp*&*pull* (Figure 9B): again the two PCs progressed similarly, with a progressive phase advance to a more pronounced peak (non-monotonous component) of PC1_EMG_ over PC1_KIN_. Note that the range of PC_KIN_ (i.e., its temporal weighting) during reach was increased by about a factor of 3 compared to *Grasp*&*pull*. Nonetheless, in both cases there was a strong correlation between those two PC1 (*r* = 0.95, *p* < 0.001 and *r* = 0.95, *p* < 0.001) for the *Reach* and *Grasp*&*pull* period, respectively. See Supplementary material for similar phase plane analysis of PC2_EMG_-PC2_KIN_ and PC3_EMG_-PC3_KIN_.

Furthermore, since grip force increase and handle displacement are functionally important during the *Grasp*&*pull* period, we also computed the phase plane between PC1_KIN_ and grip force, and that between PC1_KIN_ and displacement (shown in Figures 9C,D). PC1_KIN_ vs. grip force (*r* = 1.0, *p* < 0.001) expressed a strict correlation without time-lag between joint kinematics represented by PC1_KIN_ and the resulting time-varying grip force during grasp, suggesting a biomechanical linkage. In contrast, PC1_KIN_ vs. displacement showed, as expected, a phase advance of PC1_KIN_. Figure 9E shows a highly correlated evolution over time between PC1_EMG_ and grip force (*r* = 0.96, *p* < 0.001). This time, and in contrast to PC1_KIN_, PC1_EMG_ showed a progressive phase advance over grip force, as would be expected. A far greater phase advance of PC1_EMG_ (and consequently smaller correlation, *r* = 0.69, *p* < 0.003) was observed with respect to handle displacement (Figure 9F), indicating a tighter coupling of PC1_EMG_ to grip force than to displacement.

## Discussion

The main goal of our study was to examine how combined kinematic and kinetic grip constraints are reflected in kinematic synergies and in muscle synergies. This was investigated during the three epochs of a reach-grasp-and-hold task using PCA. Kinematic constraints were experimentally varied by imposing two different grip types, and kinetic constraints were varied through grasps (grip force) against two different loads. We provide three new key findings (i) Kinematic and muscle synergies can simultaneously accommodate combined kinematic and kinetic parameters. (ii) Upcoming grip type and load force during grasp are already represented in kinematic and muscle synergies during reach. (iii) The principal muscle synergy (PC1_EMG_) is linked (correlated) to the main kinematic synergy (PC1_KIN_) during reach and grasp. These results, taken together, suggest that synergies of muscular activation are at least in part at the origin of kinematic synergies during upper limb reach-and-grasp movements. We will first discuss the task-related kinematic properties and the EMG activations, then the separate findings of the PCA in the kinematic domain, and of the PCA in the EMG domain. Subsequently, the new findings will be put into context for the simultaneous coding of kinematic and kinetic constraints in their respective PCs, for the relation of PCs in successive epochs of the task, and for the link between muscle synergies and kinematic synergies.

### Task-related kinematic and EMG properties during hold

Inducing task-related changes in the grip configuration (kinematics) and in EMG activity was the goal of the behavioral setup. The analysis of the static joint angles as a function of task condition showed that side and precision grip constraints induced significant changes in grip configuration during the hold period. Surprisingly, the load condition also induced (smaller but significant) changes in some joint angles, although the task was considered isometric during the hold period. These load-dependent angular variations might have taken place during the grasp&pull period and persist during hold (see below). Nonetheless, these small joint variations are consistent with skin and soft tissue compliance at the fingertips under the two different load conditions (Friedman et al., 2008). This indicates that the hold period was close to, but not strictly isometric.

Furthermore, the different task conditions also provoked changes in EMG activity: the two grip types and load conditions induced significantly different EMG activity during hold. Therefore, the necessary preconditions for the subsequent PCA of the kinematics and of the EMGs were met.

### Kinematic synergies during reach, grasp, and hold

Looking at the kinematics separately, our findings are compatible with several previous studies on the human upper limb showing that combinations of a small number of kinematic synergies allows for the reconstruction of the entire set of kinematics (Santello et al., 1998; Thakur et al., 2008; Touvet et al., 2014). Most studies have looked separately at either static situations (hand posture, e.g., Santello et al., 1998; Touvet et al., 2014) or dynamic situations, such as reach-to-grasp (Santello and Soechting, 1998; Mason et al., 2001) or manual exploration (Thakur et al., 2008). Our results that kinematic synergies during reach, during grasp, and during hold account for grip type are consistent with these previous studies. During reach, this corresponds to the well-known phenomenon of hand pre-shaping (Paulignan et al., 1990; Santello and Soechting, 1998) and is consistent with results of Santello et al. (1998) and Mason et al. (2001) demonstrating correlated (synergistic) variations of joint angles during pre-shaping. For grasp, we are not aware of any previous data on time-resolved PCA on kinematics: we show that kinematic synergies exist, even though the joint variations are small during object contact. This suggests that residual joint variations during object contact are also correlated, do not represent postural noise and depend on grip type. For hold, our data are consistent with those of Santello et al. (1998) and Touvet et al. (2014), showing a substantial reduction of kinematic DoFs in PC space for different grip postures. However, our results indicate that in addition to grip type, also grip load has a significant effect on the kinematic synergies, and this during reach (c.f. Zaepffel and Brochier, 2012), grasp and hold (Table 2, see Conjoint Representation of Kinematic and Kinetic Parameters through Synergies).

### Muscle synergies during reach, grasp, and hold

There are two different ways of exploring EMG activity: our PCA of EMG activity captured the (time-varying or static) variation of EMG amplitude as a function of the independent task-variables. The resulting synergies express task-related EMG covariation, i.e., relative to grip type and load condition. These *principal muscle synergies* can (at least in theory) occur independently of any common input to the different muscles. In contrast, *pair-wise muscle synergies*, defined by pair-wise correlation of trial-by-trial EMG variability, point to common (synchronous) last-order synaptic input to two MN pools (originally suggested by Kirkwood and Sears, 1978). Two potential forms exists: (i) “coactivation,” i.e., positive correlations, where above average activity in one muscle is accompanied by above average activity in the other muscle. (ii) “reciprocal” activation (neg. correlation) where above average activity in one goes with below average activity in the other muscle (Maier and Hepp-Reymond, 1995b; Weiss and Flanders, 2004).

The existence of *principal muscle synergies* has been shown previously, such that the whole set of recorded EMGs can be approximated with relatively few muscle synergies adequately scaled and timed (for arm movements: Muceli et al., 2010; Roh et al., 2012; d'Avella and Lacquaniti, 2013; for hand/digit movements: Santello et al., 1998; Weiss and Flanders, 2004; Ajiboye and Weir, 2009). For the reach period our results are in accordance with those of Overduin et al. (2008), for grasp they are qualitatively similar to those of Brochier et al. (2004), and for hold with those of Castellini and van der Smagt (2013).

Under static conditions during hold, *pair-wise muscle synergies* were present: this is consistent with previous findings in force control during precision grip (Maier and Hepp-Reymond, 1995b) and during different grip postures (Weiss and Flanders, 2004). Coactivation occurred more frequently during precision grip than side grip, and among pairs of extrinsic muscles rather than among extrinsic-intrinsic pairs. This is coherent with the finding of stronger motor unit synchrony across motor units of extrinsic than of intrinsic hand muscles (Winges et al., 2008) and with stronger EMG-EMG coherence in extrinsic muscle pairs compared to extrinsic–intrinsic pairs (Poston et al., 2010). The presence of pair-wise muscle synergies suggests that muscle synergies are in part generated by common input to two (or more) MN pools.

### Conjoint representation of kinematic and kinetic parameters through synergies

Investigating whether synergies can account for combined kinematic and kinetic grip constraints was the main rationale of this study. We showed that the EMG PC-space of hand muscles represented indeed kinematic (grip type) together with kinetic (load force) parameters during prehension. This was also the case for the PC-space of finger kinematics.

For kinematic synergies, this held for all three periods: during reach, grasp and static hold. This is in line with the modulation of kinematic synergies observed in whole body movements depending on kinematic constraints (Berret et al., 2009), as well as on kinetic factors (Vernazza-Martin et al., 1999; Casellato et al., 2012).

For muscle synergies the conjoint representation held for the first two periods, but not during static hold, where only load force was coded for. A speculative reason for the absence of grip type coding in muscle synergies during static hold may relate to the increased mechanical constraints during this period: the kinematic chains of the index and thumb are closed by the contact with the object. Under these circumstances, changes in EMG activity will primarily affect force, not the joint configuration, as suggested by the absence of a grip type effect on the principal muscle synergies during hold. This also suggests that maintaining the grip configuration under these mechanically constrained and stable conditions does not require specific muscle activations.

### PC-space: comparison between behavioral periods—reach vs. grasp&pull vs. hold

In order to attempt a comparison between PC-spaces in the *Reach*, in the *Grasp*&*pull* and *Hold* phase, we put forward the following two hypotheses: (**H**_1_) The EMG PC-space should show load sensitivity during *Grasp*&*pull* and during *Hold* (since the hand applied forces during these two periods only), but not during *Reach*. (**H**_2_) The kinematic PC-space should not show any load sensitivity during *Reach* (since the hand was not yet in contact with the handle), neither during *Hold* (since the two static grip types were considered isometric). In contrast, the kinematic PC-space should show load sensitivity during *Grasp*&*pull*, since grip force increase and pull cannot be considered isometric.

With respect to (**H**_1_) on the representation of kinetic variables in the EMG PC-space, our data confirmed this relation only partially: as expected the load condition was accounted for during *Grasp*&*pull* and *Hold* of PC_EMG_, however, it was already present during *Reach*. This was surprising since grip force cannot be applied during reach, i.e., prior to object contact. We interpret this result as an anticipatory (reach-related) EMG activity of an upcoming (grasp and hold) load condition. This would be consistent with the observed difference in reach duration as a function of load. (**H**_2_) on the absence of kinetic representations in the kinematic PC-space during *Reach* and *Hold*: our results falsify this prediction in that load condition was accounted for in these two epochs of PC_KIN_. For the *Hold* period this can be explained by the fact that the joint configuration differed partially but significantly across loads, indicating that the task was not fully isometric (see above). However, the load-dependence during *Reach* remains surprising. Nonetheless, this kinematic dependence may be the consequence of our previously observed load-dependence of the EMG PC-space during *Reach*. It is more generally in line with data showing anticipatory kinematic changes during reach, depending on task requirement after grasp (Ansuini et al., 2008). According to our hypothesis, the load-dependence of the kinematic PC-space was confirmed during *Grasp*&*pull*.

Together our results suggest a gradual transition of the control mode from *Reach*, to *Grasp*&*pull* and *Hold*, in that the control of load seems to become increasingly more important. However, this seems to be implemented differently for kinematics and for EMG activity (Table 2). The kinematic synergies show a trend to increased importance of load force over the three epochs: a single PC dependent on GRIP and LOAD during Reach (preshaping), whereas an independent PC coding for LOAD (PC3) is present in addition during *Grasp*&*pull* (adjustment of grasp configuration and grip force increase) and *Hold*. For muscle synergies, a similar gradient toward more importance of force control over time is suggested by the presence of independent PCs controlling load in all three epochs, but the absence of a PC dependent on kinematics during hold.

In summary, our results suggest (i) that the reach phase carries anticipatory information (in terms of kinematics and of EMG) not only of the upcoming grasp configuration but also on the upcoming grip force, and (ii) that there might be gradual transitions in the control mode across the three epochs.

### Relation between kinematic and muscle synergies

If we assume that kinematic synergies are, to a significant part, a consequence of muscle synergies, then the following hypotheses can be put forward on the relation between the two: (**H**_3_) on existence: kinematic and muscle synergies must be present simultaneously; only then can muscle synergies potentially act on kinematic synergies. This should hold for all three epochs in our task. (**H**_4_) on causal relation: if the kinematic space and EMG space are somehow linked, then we would expect that the time-varying aspects (temporal weighting) of PC_EMG_ would lead those of PC_KIN_. This should hold at least for the first PC since it explains the main variance in the two spaces. This prediction follows from the causal relationship between EMGs and kinematics: it has been repeatedly shown that muscle activity leads mechanic muscle action (e.g., Johansson et al., 1994). (**H**_5_) The load sensitivity of the EMG space should be higher than that of the kinematic space. This assumption is based on two elements: first, kinetics and kinematics are dissociated under strict isometric conditions. However, our ANOVA showed that this dissociation was not strict: 2 (out of 11) kinematic DoFs varied as a function of the load during *Hold*. Second, under static conditions EMG activity is directly related to grip force production (Maier and Hepp-Reymond, 1995a).

Our results confirm the first two hypotheses: (**H**_3_) On the simultaneous existence of PCs in the kinematic and in the EMG domain: this was indeed the case. Furthermore, we found that the number of PCs to explain a similar variance was higher in the EMG PC-space than in the kinematic PC-space, i.e., #PC_EMG_ > #PC_KIN_. This was the case during *Reach* (5 PC_EMG_ vs. 3 PC_KIN_ for explaining ≥90%), during *Grasp*&*pull* (4 vs. 2), but not during *Hold* (3 vs. 3). This is consistent with the higher degree of motor redundancy in the EMG manifold compared to the kinematic DoFs (e.g., for the elbow: Buchanan et al., 1986). This trend was seen even though the set of muscles acting on the thumb, index finger and wrist was under-sampled: further differential activation of non-sampled muscles would tend to increase #PC_EMG_. (**H**_4_) On the temporal, potentially causal relation between PC_EMG_ and PC_KIN_: we used phase plane plots (as did Mason et al., 2001 within the kinematic domain only) to investigate this issue: as predicted, the main muscle synergy (PC1_EMG_), as expressed by its temporal weighting, was not independent of the main kinematic synergy (PC1_KIN_), even though the two PC-spaces were calculated fully independently. PC1_EMG_ evolved in correlation and in phase advance with PC1_KIN_ during *Reach* and during *Grasp*&*pull*. A phase advance of the muscle synergy and a correlated temporal evolution with the kinematic synergy would be expected if there were a causal relation between the two. This is consistent with the hypothesis that muscle synergies are, at least partially, at the source of kinematic synergies. However, (**H**_5_) on the higher load sensitivity of PC_EMG_ compared to PC_KIN_ was only partially confirmed. A first indicator confirmed this prediction: the ANOVA (Table 2) showed that PC_EMG_ had consistently higher and significant *F*-values (lower *p*-values) for load condition compared to PC_KIN_. However, a second indicator, the phase plots, did not confirm this hypothesis: PC1_EMG_ against grip force showed, as predicted, a high correlation, but PC1_KIN_ showed an even higher and perfect correlation with grip force, i.e., linear and without lag. In fact, this perfect correlation should have been expected, given that the task was non-isometric during *Grasp*&*pull*: in hindsight, this strict correlation represents the *mechanic* interaction between changes in joint angles (represented by PC1 to 78%), small but non-negligible displacement of force sensors and resulting grip force during *Grasp*&*pull*. This interaction between PC_KIN_ and grip force is further mediated by skin and joint compliance (Friedman et al., 2008).

It is an open question whether second-order PCs can or should be related among EMG and kinematics PC spaces. In any case, muscle synergies might not be directly or linearly related to movement kinematics, since movements and forces are produced through joint torques. The synergistic relation between EMG and torque has recently been investigated for reach movements (d'Avella and Lacquaniti, 2013). Nonetheless, our data is consistent with the hypothesis, at a first-order approximation, of a causal relation between muscle synergies and kinematic synergies.

### On the neural substrate of synergies

Disentangling biomechanical components from neural components in the generation of kinematic synergies remains problematic. Biomechanical components, such as muscles acting on several joints within one digit and others acting across several digits, lead to limited individuation of digit movements (Lang and Schieber, 2004). Neural components then superimpose their action on these biomechanical constraints. Our results stress the importance of neural drive (captured by the EMG) in the generation of kinematic synergies. However, muscular activations need to be considered on two levels: one “principal muscle synergies”) is related to systematic variations of EMG activity (the time-varying profile) as a function of task constraints, the other (“pair-wise muscle-synergies”) relates to simultaneous covariation (coactivation) of multiple muscles. The neural substrate of the former resides in cortical (Davare et al., 2011) and subcortical (Prodoehl et al., 2009; Manto et al., 2012) structures and relates to voluntary control of the upper limb, the latter represents common input to spinal MN pools (Bennett and Lemon, 1996; Santello et al., 2013). To date, it remains open whether the presence of synergies indicates an organizational principle, or whether they simply reflect task constraints. Although data consistent with a neural control of the upper limb through synergies has been reported (in the non-human primate: Holdefer and Miller, 2002; Takei and Seki, 2013; Kirsch et al., 2014; in humans: Berger and d'Avella, 2014), there is no direct evidence that this is the case (Mollazadeh et al., 2014) and how it would eventually be implemented.

## Conclusions

We have shown that kinematic synergies of the hand during a reach-grasp-and-hold task can simultaneously accommodate combined kinematic (grip type) and kinetic (grip force) task constraints. This was also the case for muscle synergies. Furthermore, our results suggest that the reach phase carries anticipatory information (in terms of kinematics and of EMG) not only on the upcoming grasp configuration, but also on the upcoming grip force. Finally, we found some systematic relations between kinematic synergies and muscle synergies consistent with the hypothesis that EMG synergies are, at least partially, at the source of kinematic synergies.

### Conflict of interest statement

The authors declare that the research was conducted in the absence of any commercial or financial relationships that could be construed as a potential conflict of interest.
