# Supplementary material for: Differences between kinematic synergies and muscle synergies during two-digit grasping
Source: Front Hum Neurosci. 2015 Mar 26;9:165. doi: 10.3389/fnhum.2015.00165 (PMC4374551; doi:10.3389/fnhum.2015.00165)
Supplement: Supplementary file 1 [file Image1.PDF]

Supplementary Material

Differences between kinematic synergies and muscle synergies during two-digit grasping

Michele Tagliabue[1], Anna-Lisa Ciancio[2], Thomas Brochier[3], Selim Eskiizmirliler[1,4], Marc A. Maier[1,4]*

[1]Neuroscience Research Federation FR3636, CNRS, Université Paris Descartes, Paris, France
[2]Laboratory of Biomedical Robotic and Biomicrosystem, Università Campus Bio-Medico di Roma, Roma, Italy
[3]Institut de Neurosciences de la Timone, UMR 7289, CNRS, Aix-Marseille Université, Marseille, France
[4]Université Paris Diderot, Sorbonne Paris Cité, Paris, France

*** Correspondence:** Marc Maier, FR3636 Neurosciences, Université Paris Descartes, 45 rue des Saints Pères, 75006 Paris, France.
e-mail: marc.maier@parisdescartes.fr

1. Supplementary Figure

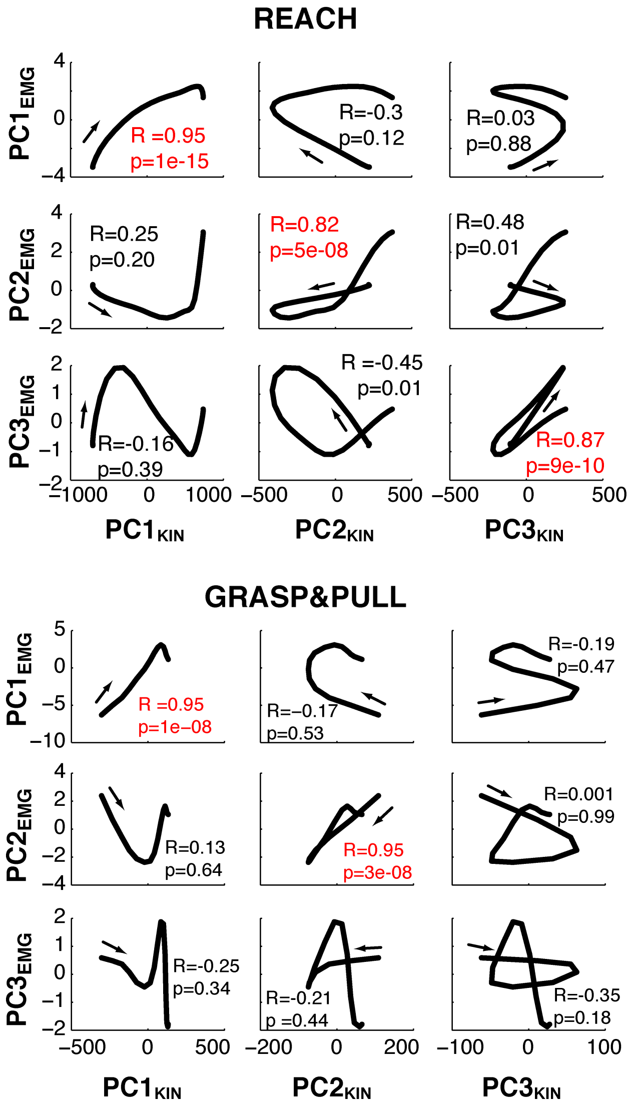

Supplementary Figure 1. Phase plots of the temporal weighting from the first three PCs of the EMG manifold (PC_{EMG}) against those of the PCs from the kinematic manifold (PC_{KIN}). All 9 combinations of PC1-3_{EMG} and PC1-3 $_{KIN}$ are shown for the reach period (top) and for the grasp&pull period (below). Correlation coefficients are given for each pair (in red if $p < 0.001$). Among the 9 potential combinations of the first three PCs, only $PC1_{EMG}$-$PC1_{KIN}$, $PC2_{EMG}$-$PC2_{KIN}$ and $PC3_{EMG}$-$PC3_{KIN}$ showed significant correlations during reach, but none among the non-corresponding PCs (e.g. $PC1_{EMG}$-$PC3_{KIN}$). For the grasp&pull period this held for $PC1_{EMG}$-$PC1_{KIN}$ and $PC2_{EMG}$-$PC2_{KIN}$.
